# EEG dataset for energy data visualizations

**DOI:** 10.1016/j.dib.2023.109933

**Published:** 2023-12-10

**Authors:** Omer Faruk Kucukler, Abbes Amira, Hossein Malekmohamadi

**Affiliations:** a Institute of Artificial Intelligence, De Montfort University, Leicester, UK; b Department of Computer Science, University of Sharjah, Sharjah, UAE

**Keywords:** Electroencephalography, Energy efficiency, Data visualization, Brain-computer interface, Human-computer interaction, Generative adversarial networks

## Abstract

User behavior plays a substantial role in shaping household energy use. Nevertheless, the methodologies employed by researchers to examine user behavior exhibit certain limitations in terms of their reach. The present article introduces an openly accessible collection of electroencephalography (EEG) recordings, comprising EEG data collected from individuals who were subjected to energy data visualizations. The dataset comprises EEG recordings obtained from 28 individuals who were in good health. The EEG recordings were collected using a 32-channel EMOTIV EEG device, and the international 10-20 electrode system was employed for precise electrode placement. The energy data visualizations were generated and showcased utilizing the PsychoPy software. To ascertain the participants' affective state, they were requested to rate the valence and arousal of each stimulus through the utilization of a self-assessment manikin (SAM). Additionally, three inquiries were posed for every stimulation. The dataset includes both original data visualizations and ratings. Additionally, the raw EEG data has been divided into segments consisting of data visualizations and neutral images, with the use of event markers, in order to assist analysis. The EEG recordings were recorded and stored utilizing the EMOTIVPro application, whereas the subjective reactions were captured and preserved using the PsychoPy application. Furthermore, the generation of synthetic EEG data is accomplished by employing the Generative Adversarial Network (GAN) architecture on the acquired EEG dataset. The synthetic EEG data created is integrated with empirical EEG data, and afterwards subjected to qualitative and quantitative analysis in order to improve performance. The dataset presented herein showcases a pioneering utilization of EEG investigation and offers a valuable foundation for scholars in the domains of computer science, energy conservation, artificial intelligence, brain-computer interfaces, and human-computer interaction.

Specifications Table

**Table:** 

Subject	Human-Computer Interaction
Specific subject area	Analysis of energy consumption patterns using electroencephalographic (EEG) signals
Data format	Raw EEG data, synthetic EEG data and augmented EEG data
Type of data	Raw EEG data (.csv or .rar format) Artificial EEG data (.csv or .rar format) SAM ratings and answers to questions (.csv or .rar format)
Data collection	EEGs were recorded utilizing a 32-channel wireless EMOTIV EPOC Flex gel kit, with a sampling rate of 128 Hz, specifically targeting the 10-20 electrode locations. The data collection process involved the utilization of the EMOTIVPro software. Energy data visualizations are employed as stimuli. The Pavlovia program, which serves as a platform for conducting PsychoPy studies, was utilized to present the participants with the generated stimuli. In addition, Generative Adversarial Networks (GANs) are employed for the purpose of generating synthetic data. Twenty-eight participants had their EEGs recorded while being presented with visual representations of energy data. In a bright room, each participant sat at a desk with a computer in front of them. Participants had an EEG cap placed on their heads, and data was recorded from the caps' sensors via a wireless connection to a laptop. The EEG signal was annotated with the aid of PsychoPy's integration with EMOTIVPro. Furthermore, empirical EEG samples are used to generate synthetic data for all channels.
Data source location	• Institution: De Montfort University • City/Town/Region: Leicester • Country: United Kingdom
Data accessibility	Repository name: EEG Dataset Collected During Energy Data Visualization Stimuli Presentation (EDAVIS) Data identification number: 10.17632/w9nk4mvgbb.3 Direct URL to data: https://data.mendeley.com/datasets/w9nk4mvgbb

## 1 Value of the Data

• The dataset comprises EEG recordings pertaining to various visualizations of energy data. The distinguishing feature of the open-access energy data visualization (EDAVIS) EEG dataset is its inclusion of a diverse range of graph stimuli representing energy consumption.
• This dataset serves as a foundational resource for exploring a novel research domain focused on discerning the energy consumers' inclinations towards energy data visualization.
• Synthetic data has the potential to augment the size of existing datasets and contribute to the enhancement of classification performance.
• The dataset holds potential benefits for students and researchers alike, as it can be utilized for the purpose of training machine learning models and other applications within the domains of neuroscience, computer science, and data science.
• The dataset has potential applications in various domains, including signal processing techniques such as feature extraction, conversion, and pre-processing. Additionally, it can be utilized for human behavior analysis, specifically in the examination of EEG features alongside subjective responses. Furthermore, the dataset holds relevance in the field of machine learning, particularly in the classification of emotions.

## 2 Data Description

The dataset includes two data containers (1) raw EEG data for energy data visualizations and (2) augmented data for energy data visualizations. The data format is presented as tables in .csv format or compressed zip files.

### 2.1 Objective

Data visualizations are an effective instrument for presenting the requisite information to analyze consumer behavior and proficiently monitor energy consumption [1]. The inclusion of specific visualizations and data types is of utmost importance when employing data visualizations to analyze energy consumption [2], [3], [4]. The primary focus of this dataset is the analysis of participant behavior towards data visualization, rather than the prediction of consumption patterns for the purpose of enhancing consumption control, as explored in previous studies [5,6]. Moreover, the utilization of synthetic EEG data can prove to be a valuable instrument in augmenting the analysis and results of associated task accomplishment [7,8]. Furthermore, the dataset presents a novel resource for researchers investigating the advancement of machine learning models in the field of behavior analysis and other disciplines within data science.

### 2.2 Raw Data: EEG data for Energy Data Visualizations

The dataset encompasses data collected from a sample of 28 participants who were exposed to a series of 10 distinct data visualizations and neutral images during a single task. The positioning of the electrodes adheres to the established 10-20 electrode system. Emotion recognition is consistently linked to the frontal cortex [9], [10], [11]. In this context, the EEG channels that are taken into consideration include Fp1, Fp2, F3, F4, F7, and F8. The EEG data is recorded and stored in the comma-separated values (.csv) format. Table 1 provides a comprehensive overview of the specific details pertaining to the EEG data file.

**Table 1:** Definition of dataset files.

Data visualization	Subject	Channel	Valence	Arousal
1	1Sub - 28Sub	Fp1, Fp2, F3, F4, F7 and F8	HV or LV	HA or LA
0 (Neutral)	1Sub - 28Sub	Fp1, Fp2, F3, F4, F7 and F8	-	-
2	1Sub - 28Sub	Fp1, Fp2, F3, F4, F7 and F8	HV or LV	HA or LA
0 (Neutral)	1Sub - 28Sub	Fp1, Fp2, F3, F4, F7 and F8	-	-
3	1Sub - 28Sub	Fp1, Fp2, F3, F4, F7 and F8	HV or LV	HA or LA
0 (Neutral)	1Sub - 28Sub	Fp1, Fp2, F3, F4, F7 and F8	-	-
4	1Sub - 28Sub	Fp1, Fp2, F3, F4, F7 and F8	HV or LV	HA or LA
0 (Neutral)	1Sub - 28Sub	Fp1, Fp2, F3, F4, F7 and F8	-	-
5	1Sub - 28Sub	Fp1, Fp2, F3, F4, F7 and F8	HV or LV	HA or LA
0 (Neutral)	1Sub - 28Sub	Fp1, Fp2, F3, F4, F7 and F8	-	-
6	1Sub - 28Sub	Fp1, Fp2, F3, F4, F7 and F8	HV or LV	HA or LA
0 (Neutral)	1Sub - 28Sub	Fp1, Fp2, F3, F4, F7 and F8	-	-
7	1Sub - 28Sub	Fp1, Fp2, F3, F4, F7 and F8	HV or LV	HA or LA
0 (Neutral)	1Sub - 28Sub	Fp1, Fp2, F3, F4, F7 and F8	-	-
8	1Sub - 28Sub	Fp1, Fp2, F3, F4, F7 and F8	HV or LV	HA or LA
0 (Neutral)	1Sub - 28Sub	Fp1, Fp2, F3, F4, F7 and F8	-	-
9	1Sub - 28Sub	Fp1, Fp2, F3, F4, F7 and F8	HV or LV	HA or LA
0 (Neutral)	1Sub - 28Sub	Fp1, Fp2, F3, F4, F7 and F8	-	-
10	1Sub - 28Sub	Fp1, Fp2, F3, F4, F7 and F8	HV or LV	HA or LA
0 (Neutral)	1Sub - 28Sub	Fp1, Fp2, F3, F4, F7 and F8	-	-

**Fig. 1:**
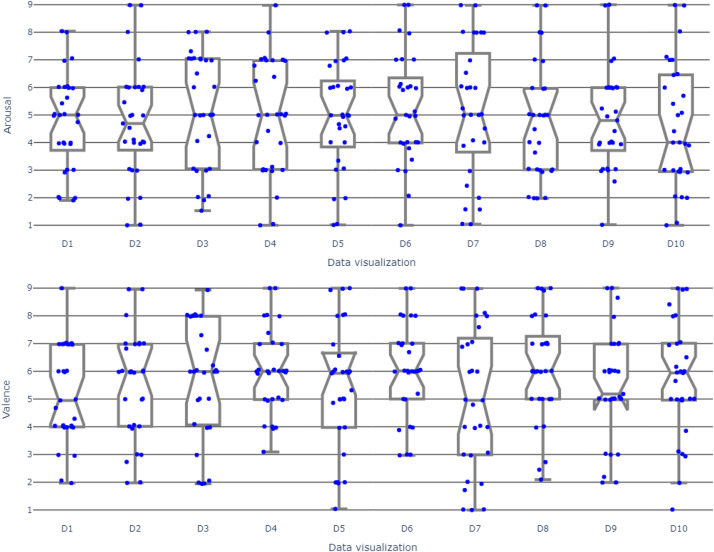
Valence and arousal ratings from all subjects for each data visualization.

The dataset files are categorized into two distinct folders. The folder containing the EEG data comprises recordings in .csv format, specifically pertaining to EEG data obtained from six channels and artificial data. Additionally, the .csv folder contains data related to visualisation numbers, valence class, arousal class, and subjects. The classification of the class is determined by utilizing a rating value of 5 as the central point. Ratings of 5 and above are categorized as high (H), while ratings below 5 are categorized as low (L) on a rating scale ranging from 1 to 9. In addition to the provision of rating scores, the customization of classes can be achieved through the utilization of various thresholds. The event markers serve the purpose of extracting data visualizations and neutral images, and they are not encompassed within the file. The folder contains the original data visualizations utilized in the experiments, presented in .png format. The subjective response folder encompasses both the Self-Assessment Manikin (SAM) ratings [12] and the participants' responses to questions for each data visualisation. Tables 2 and 3 present the mean values of SAM ratings for valence and arousal, as well as the ratings for the questions. Fig. 1 presents the distribution of valence and arousal ratings for data visualizations. EEG data and subjective responses location in the repository is illustrated below:

**Table 2:** SAM ratings for each data visualization stimulus.

Data visualization	Average Valence Responses	Average Arousal Responses
1	4.95	4.52
2	5.48	5.59
3	5.48	4.77
4	6.41	4.91
5	5.56	5.15
6	5.77	4.49
7	4.57	4.93
8	5.81	4.85
9	5.97	5.08
10	6.14	4.93

**Table 3:** The answers to the questions for each data visualization.

Data visualization	Average answers for Q1^a^	Average answers for Q2^b^	Average answers for Q3^c^
1	3.00	2.89	2.82
2	3.11	2.93	2.96
3	2.96	2.82	2.89
4	4.04	4.36	3.21
5	2.93	3.11	2.86
6	2.96	2.96	2.75
7	2.46	2.64	2.29
8	3.25	3.54	3.18
9	3.64	4.18	2.79
10	3.71	3.64	3.11

a How effective was the power consumption visualization?

b Was the graph's information clear to understand?

c Do you find the presented visualization aesthetically appealing?

EEG data location in the repository folders:

root>EEG data
  >>EEGdataset.csv
  >>additionally data visualizations in .png format

Subjective responses location in the repository folders:

root>Subjective response
  >>SAM ratings and question answers.csv

Table 2 presents the mean valence and arousal ratings obtained from the entire participant pool for ten distinct data visualizations. Values below 5 are indicative of a low-class status, whereas values above 5 are indicative of a high-class status. Furthermore, for each of the ten data visualizations, a set of three inquiries is posed, and the mean ratings for all participants are presented in Table 3. The responses provided by participants reflect their level of comprehension of the presented information through the use of data visualizations, with a rating scale ranging from 1 (indicating the lowest level of understanding) to 5 (indicating the highest level of understanding). The effectiveness of the questionnaire is evaluated using reliability statistics. The Cronbach's alpha calculated as 0.814 which delivers a promising internal reliability.

### 2.3 Augmented Data: EEG data for Energy Data Visualizations

The provided dataset encompasses augmented EEG data specifically collected for energy data visualization stimuli. The repository's “Artificial EEG Data” folder encompasses samples that have been augmented with synthetic data as well as those that have been generated artificially. “Valence” and “Arousal” are two emotion folders contained within this directory. Thereafter, individual folders for each channel are created within the emotion folders. The dataset comprises three discrete sample groups, namely mixed, male, and female. In addition, one thousand synthetic samples that have been produced are included. The location of one channel data folder within the repository, containing various samples, is illustrated below:

Folders that symbolize data pertaining to the subsequent categories:

root> Artificial EEG data
  >>Arousal
  >>>Fp1
    - Male synthetic data (1,000 samples)
    - Female synthetic data (1,000 samples)
    - Mixed synthetic data (1,000 samples)
    - Male empirical data
    - Female empirical data
    - Mixed empirical data
    - Male augmented data (50 samples and 1,000 samples)
    - Female augmented data (50 samples and 1,000 samples)
    - Mixed augmented data (50 samples and 1,000 samples)
    - Test data (Not used in generation)

## 3 Experimental Design, Materials and Methods

### 3.1 Empirical EEG Data Collection for Energy Data Visualizations

**Fig. 2:**
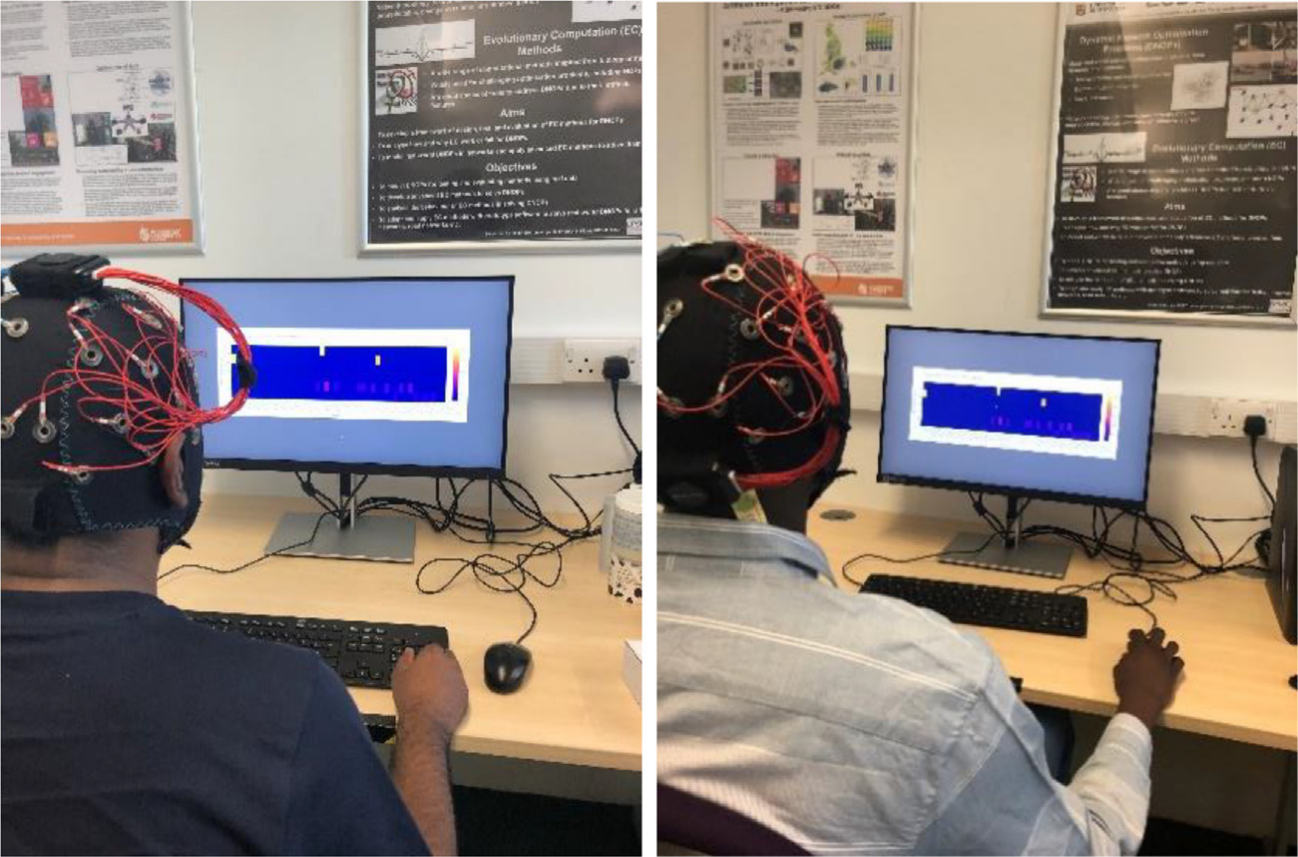
Participants in the experiment.

The first step in the process of experimental design entails the creation of visual representations of energy data. The initial step involves the generation of visualizations utilizing the openly accessible dataset referred to as UK-DALE [13]. Ten unique data visualizations are produced by utilizing samples extracted from the dataset. To procure EEGs, a team of researchers obtained an EMOTIV EPOC Flex EEG cap. The selected configuration of this equipment is a gel kit consisting of 32 channels, with a standardized dimension of 56 cm. Another component of the experiment involves the stimuli. The stimuli are prepared and presented utilizing the PsychoPy platform [14], which additionally incorporates the transmission of event markers. During the course of the experimental protocol, the participants were initially subjected to the presentation of a data visualisation, which was subsequently followed by the display of a neutral image. Following that, participants were instructed to provide subjective ratings by evaluating their perceived levels of valence and arousal. Ultimately, the participants were obligated to provide answers to a set of inquiries. The aforementioned protocol is consistently followed for each occurrence of data visualisation. The concept of the neutral image, represented by an empty page, is widely recognized as a primary stimulus that reduces emotional arousal in anticipation of the following image. The process in PsychoPy is both intentionally designed and effectively implemented. To ensure effective communication with participants, a timer has been integrated to facilitate the process of SAM ratings and question slides. The valence and arousal ratings are systematically categorized on a numerical continuum spanning from 1 to 9, operating as independent constructs. The assessment of each question's rating is established based on the participants' responses, utilizing a 5-point scale for measurement. The participants are given instructions to disclose personal information on the first page of the stimuli, accompanied by a guarantee that their identities will be protected. The following section presents succinct instructions to be followed before initiating the experiment. The commencement of the experiment is facilitated by depressing the space bar, as specified on the instructional page. Fig. 2 illustrates a group of participants who participated in the experimental investigation. The research involved a cohort of 28 participants, consisting of 23 males and 5 females. The researchers computed the average age of the participants to be 27.8. Participants invited through the posters around faculties of De Montfort University and some leaflets are prepared to invite people for the study. A small amount of reward is proposed for participation as a voucher. The study encompasses a heterogeneous cohort, with no requirement for a specific medical condition as a prerequisite for inclusion. Before the initiation of the experiment, all participants were required to provide informed consent by signing a consent form, thereby granting authorization for the utilization of their data. The current study has obtained ethical approval from the research ethics committee at De Montfort University with the reference number of 421051.

**Fig. 3:**
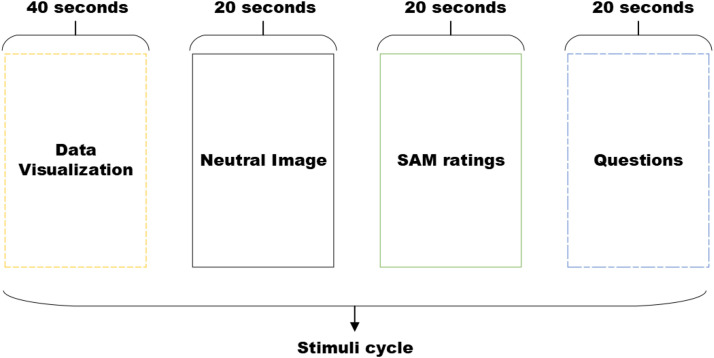
Procedure of stimuli.

Before initiating the experiment, the EEG cap is placed on the subject's scalp. The sensors are connected in a sequential manner through the application of an electrolyte gel after the sensor gaps have been cleaned using isopropyl alcohol. The EMOTIVPro software is employed for the purpose of establishing and validating the connections of sensors, thereby ensuring a level of quality that attains a 100% threshold. The individuals are situated in close proximity to a computer screen in a setting that is adequately illuminated. Upon the completion of the requisite preparations, the commencement of the experiment takes place. The information pertaining to the duration and sequence of the stimulus is presented in Fig. 3. The experiment has a total duration of 17 minutes, during which the first-cycle SAM rating and question parts are extended to 30 seconds. The purpose of this extension is to enhance the understanding of participants regarding these tasks.

The integrity of the gathered dataset was ensured by conducting each experiment using the exact same procedures. To determine the experimental procedure and acquire variables for assessing the quality of work, a preliminary study is conducted [15]. The statistical significance of experiment stimuli is examined in this prior research, which suggests encouraging results for the primary experiments. The experimenter, having acquired experience during the data collection phase of a previous study, has made any required adjustments to the current experimental phase. For instance, participant-specific information regarding sensor connection capabilities, device sensitivity, and requisite instructions has been gathered. During the main experiment phase, participant noise generation, device control, and sensor connection checks have been under controlled. The experimenter ensured that the sensor and signal quality were both 100 percent and was prepared to intervene if necessary. During the experiment, participants were provided with concise explanations and directives, including the importance of maintaining stillness, reducing intentional blinking, and minimizing body movement. The aforementioned measures were implemented on every participant, ensuring that the experiment maintained the highest possible quality. In order to ensure uniformity, all investigations were conducted under identical laboratory conditions. Throughout the experiment, the experimenter monitored recordings and carefully examined them for inconsistencies in order to annotate the data as necessary. EEG data are preprocessed subsequent to the experiment in order to eliminate artifacts including muscle activity, eye blinks, and other sounds. In fact, as part of its internal preprocessing capability, EMOTIV EPOC Flex filters the output signal at 0.2–45 Hz with a digital 5th-order Sinc filter and at 50 Hz and 60 Hz with a digital notch filter to reduce noise [16]. EEG signals are recorded with a sampling frequency of 128 Hz. Additionally, prior to analyzing EEGs, we implemented a Butterworth band-pass filter spanning from 0.5 to 60 Hz and a notch filter at 50 Hz. In addition to other preprocessing steps, it is crucial to specify that EEG signal segments associated with each energy data visualization are extracted. As was previously stated, event markers are utilized during recording. The event markers for each data visualization are identified manually, and these segments are extracted from EEG recordings. Furthermore, recordings of neutral image stimuli are extracted and appended to the data folder. Class information is subsequently appended in conjunction with the corresponding stimulus. Class information is extracted manually from folders containing subjective responses and letter conversions are applied using a 1 to 9 scale, with 5 serving as the midpoint. Aside from that, labels ranging from 1 to 10 are assigned to each data visualization, with 0 representing neutral images. Additionally, subject identifiers are added manually to distinguish the recordings of each subject. The datasets that have been made public consist of preprocessed EEG recordings; any image conversions, segmentations, windowing, feature extraction, and statistical analysis performed on the recordings are permitted for use in further research and validation by researchers.

It is worth noting that in order to demonstrate the power of a dataset, its effectiveness and generalizability are crucial factors to consider. Effect sizes are regarded as a dependable method for demonstrating the diversity of a dataset's components in this context. The experiment in which some participants' demographic information was gathered for this investigation. Age, gender, education, occupation, background, and vision aid are some of these factors. One can employ a sensitivity analysis to examine the impact of each group within the dataset. In order to assess the effect sizes and p-values of each demographic, the one-way ANOVA statistical test is utilized. With the goal of depicting EEG signals, the mean values of each participant are utilized. Subgroups within demographics are defined by distinct parameters as displayed in Table 4. The background contains the greatest variety with 16 different areas. For statistical analysis, age is divided into three subgroups: ages 0 to 25, ages 25 to 40, and ages over 40.

**Table 4:** Subgroup division for dataset's demographics.

Demographics	Subgroup Information
Age	0 – 25, 25 – 40, and above 40
Gender	Male and Female
Education	Primary School, Secondary School, High School, BSC, MSc
Occupation	Student, Engineer, Developer, Taxi Driver, Chef, Salesman, Senior Lecturer
Background	Computer Science, Graphic Design, Electrical Engineering, Computer Games Programming, Mechanical Engineering, Architecture, Politics, Mechanic, International Business and Management, Data Science, Economics, Software Engineer, Chef, Cyber Security, Salesman, Art and Design
Vision Aid	None and Glasses

After the production of subgroups, the calculated means of EEG signals for each participant are appended to the demographics-containing dataset to conduct statistical analysis. The results of statistical tests conducted on each demographic are detailed in Table 5. Consequently, the results demonstrate that demographics influence outcomes differently. The effect size of the background on the dataset is 0.895 eta-squared, and the outcome of the ANOVA test indicates that the background is a statistically significant factor in the dataset. This indicates that datasets with greater participant diversity yield superior results. Likewise, occupation exhibits a substantial impact, as evidenced by its effect size of 0.766 and statistical significance as indicated by p-values below 0.001. These demographics demonstrate the variety of occupations and backgrounds represented in the dataset. Conversely, the effect sizes for Vision Aid and Education are small and lack statistical significance (p>0.05), indicating that these variables exert a comparatively modest influence on the final result.

**Table 5:** Sensitivity analysis results for dataset's demographics.

Demographics	Effect Sizes (eta-squared)	P values
Age	0.29	0.014
Gender	0.178	0.025
Education	0.013	0.998
Occupation	**0.766**	**<0.001**
Background	**0.895**	**<0.001**
Vision Aid	0.026	0.408

In relation to limitations, the influence of gender group on the results of the dataset is negligible. The dataset exhibits a comparatively limited representation of female participants, indicating potential for increased female representation. Participant recruitment was subject to certain restrictions, particularly with regard to females. It is noteworthy to mention that this data collection commenced during the COVID-19 pandemic, and after the pandemic, individuals, particularly women, exhibited reluctance to participate in experiments involving close proximity. One possible explanation is the experimental protocol, which involves the application of electrolyte gel and the connection of an EEG sensor to the head. Females have shown little interest in the experiment, despite its promotion through the distribution of leaflets and posters and in-person invitations. In contrast, age demographics indicate a small effect, spanning from 19 to 48 years old. Random sampling is employed to select participants, ensuring that the dataset remains relatively diverse. This is in contrast to comparable studies that focus solely on a specific type of participant subgroup, such as students, where age demographics might have a minimal influence [17]. In addition, the dataset contains a distinct cognitive assignment that employs visualizations of energy data as stimuli. A total of ten different data visualizations were employed in this research to illustrate energy consumption. A greater quantity of data visualizations could potentially enhance the efficacy of analysis.

In general, this research demonstrates distinctiveness with respect to stimuli for visualizing energy data and the examination of participants' reactions to those stimuli. Furthermore, an examination of sample size calculations in a review article emphasizes the lack of literature containing effect sizes and the power of EEG datasets [18]. Upon reviewing comparable and well-known studies related to EEG emotion recognition, we find that such details have been absent [17,[19], [20], [21]. The demographics of the dataset and the effect sizes are presented in this paper as a point of reference for future research. Subsequent investigations may augment the diversity of demographic factors (e.g., age, gender, education, and vision aid) and employ a wider array of data visualizations to rectify the shortcomings of this study.

### 3.2 Augmented Data with Generative AI: EEG Data for Energy Data Visualizations

The restricted maneuverability for classification is mostly attributed to the poor signal-to-noise ratio of EEGs. To address this issue, huge data sets are required for effective mitigation [22]. The concept of General Adversarial Networks (GANs) was introduced by the author mentioned in reference [23]. GANs have the capability to generate highly realistic samples from intricate datasets. However, EEGs are infrequently utilized in conjunction with GANs due to the relatively new development of GANs and the necessity for further investigation [8]. GANs comprise a pair of neural networks, namely the generator and the discriminator, which function as a machine learning framework. The generator is trained to produce novel samples that are indistinguishable from real samples. In the present context, the generator is employed to generate continuous samples of EEG data that encompass the underlying EEG properties associated with the task being performed. In contrast, the discriminator is responsible for determining, based on the provided data, whether it originates from the real world or has been generated synthetically by the generator. The generator will consistently generate synthetic samples in order to deceive the discriminator by making them indistinguishable from actual samples. Subsequently, it may be employed as synthetic EEG data. The utilization of augmented EEG data has demonstrated enhanced classification capabilities, as evidenced by previous studies [24,25]. Nevertheless, this application is in its nascent stage and requires further elucidation across various dimensions. This study aims to investigate methods for increasing the number of data and assess the impact on classification performance.

#### 3.2.1 Generative Adversarial Networks

The GAN is composed of two distinct neural network designs. The generator G accepts an input of a randomly sampled vector z from the probability distribution $P_{z}$ . It then computes a sample $\overset{´}{y}$ according to the following procedure:

(1) $$\overset{´}{y}=G\left(z;\theta_{G}\right)$$

where $\theta_{G}$ represents the parameters of the generator. The discriminator receives input in the form of generated samples, denoted as $\overset{´}{y}$, which are drawn from the distribution $P_{\theta }$ generated by the generator. Alternatively, it also takes in real samples, denoted as *y*, which are drawn from the distribution $P_{data}$ of a real dataset. The discriminator employs a validation process to ascertain the authenticity of the samples:

(2) $$v=D\left(\left\{\overset{´}{y},y\right\};\theta_{D}\right)$$

where $\theta_{D}$ represents the parameters of the discriminator. The validation score, denoted as v, quantifies the likelihood of the dataset's real samples. The discriminator aims to optimize the likelihood of samples by awarding validation ratings to both genuine and counterfeit samples. The generator functions by employing a formula that aims to minimize the output of the discriminator in order to generate samples that closely resemble real data:

(3) $$\frac{\text{min}}{G}\left(1-\log\left(D\left(\overset{´}{y}\right)\right)\right)$$

The game being referred to is commonly known as a minimax game [23], in which two players, namely the generator and the discriminator, engage in strategic decision-making. The training process of the GAN can be characterized by the following function:

(4) $$\frac{\min}{G}\frac{\max}{D}v\left(G,D\right)=E_{y∼P_{data}\left(y\right)}\log D\left(y\right)+E_{z∼P_{z}\left(z\right)}\log\left(1-D\left(y\right)\right)$$

We implemented an architectural framework based on the work of Willams et al. [8]. In summary, the first GAN architecture is plagued by issues of training instability and mode collapse. In order to tackle these concerns, the Wasserstein-GAN (WGAN) proposed by Arjovsky et al. in 2017 [24] is employed, wherein the initial objective function is substituted with the Wasserstein distance. The utilization of weight clipping or gradient penalty (GP) has been proposed as a means to mitigate training instability [25]. In this study, a generator and discriminator for time series (TTS) are developed using a transformer-encoder architecture, with the aim of capturing temporal relations [26]. The time data is tokenized using the patches described by Li et al. [26]. The input to the GAN comprises data pertaining to the experimental condition denoted as c, while the corresponding architecture is referred to as conditional GANs [27]. According to the literature, the conditional TTS-WGANGP architecture has been proposed as a means to attain enhanced training stability and reduced instances of collapsing [8]. Additionally, this architecture leverages the benefits offered by transformers when applied to time series data [8]. The GAN parameters provided in Table 6 were revised to incorporate a more robust calculation method for our EEG data, which encompasses a greater number of data points for each condition. This study examines pairings of situations characterized by low valence and high valence, as well as low arousal and high arousal. Each pair of conditions is trained independently for each channel. The dataset consists of six channels of EEG data, and the GAN is trained individually on each channel. The training process was executed using the ADAM optimizer, with a learning rate of 0.001. A fourth-order bandpass filter with a frequency range of 0.1Hz to 30Hz is implemented prior to the input of a discriminator. The discriminator undergoes five iterations as described in reference [8]. The window slicing technique is employed to generate samples.

**Table 6:** The GAN parameters.

Description	Value
Number of Epochs	10
Learning Rate	0.001
Batch Size	16
Patch Size	5
Total Sequence Length	50
Generated Sequence Length	30
Number of Generated Channels	1

#### 3.2.2 Methodology

In this study, we conducted an evaluation to determine the extent to which the use of GANs in augmenting EEG data resulted in the generation of realistic EEG samples that accurately represented the underlying brain properties. Subsequently, we investigated whether the incorporation of GAN-augmented EEG data had a positive impact on the performance of classification algorithms. The class-level EEG data was subjected to visual inspection, encompassing both empirical and synthetic data. A methodology was employed wherein both empirical and augmented EEG data were trained and validated using a subset of test data from six participants. This approach was utilized to assess the performance of the classifier. A GAN was utilized to train on a dataset consisting of class-level data from 22 participants. Subsequently, 1,000 samples were created for each of the valence (low-high) and arousal (low-high) classes for both analyses. In this study, a random forest classifier is employed. Three sample sizes are examined for the extent of possible augmentation. Sample sizes are adopted using data samples of males, females and mixed empirical data. Mixed empirical data consists of 22 participants, while males are 17 and females are 5. A single sample corresponds to a certain category of data, while the EEG dataset used consists of six channels of data, each of which encompasses ten distinct classes for visualizations. For example, mixed samples equal 1320 (22 participants x 6 channels x 10 data visualizations). Three GAN training sessions are carried out for these separate empirical samples. A total of 5020 data points were utilized for each class data. Classification performances are evaluated on both empirical and augmented data samples. Augmented data for three sample sizes are obtained by aggregating 1,000 samples into bins of 20 for 50 synthetic samples and combined with empirical data for related training samples. Table 7 shows sample sizes for three groups and generated total samples. In addition, a further comparison of synthetically generated data performances is evaluated using an 80% training and 20% test split procedure for three groups of synthetic data. For this calculation, all generated 1,000 samples are used from each channel.

**Table 7:** Sample sizes for GAN training.

Sample	Empirical Sample Size	Averaged Synthetic Sample Size	Augmented Sample Size
Male	1020	300	1320
Female	300	300	600
Mixed	1320	300	1620

#### 3.2.3 Results and Conclusions

**Fig. 4:**
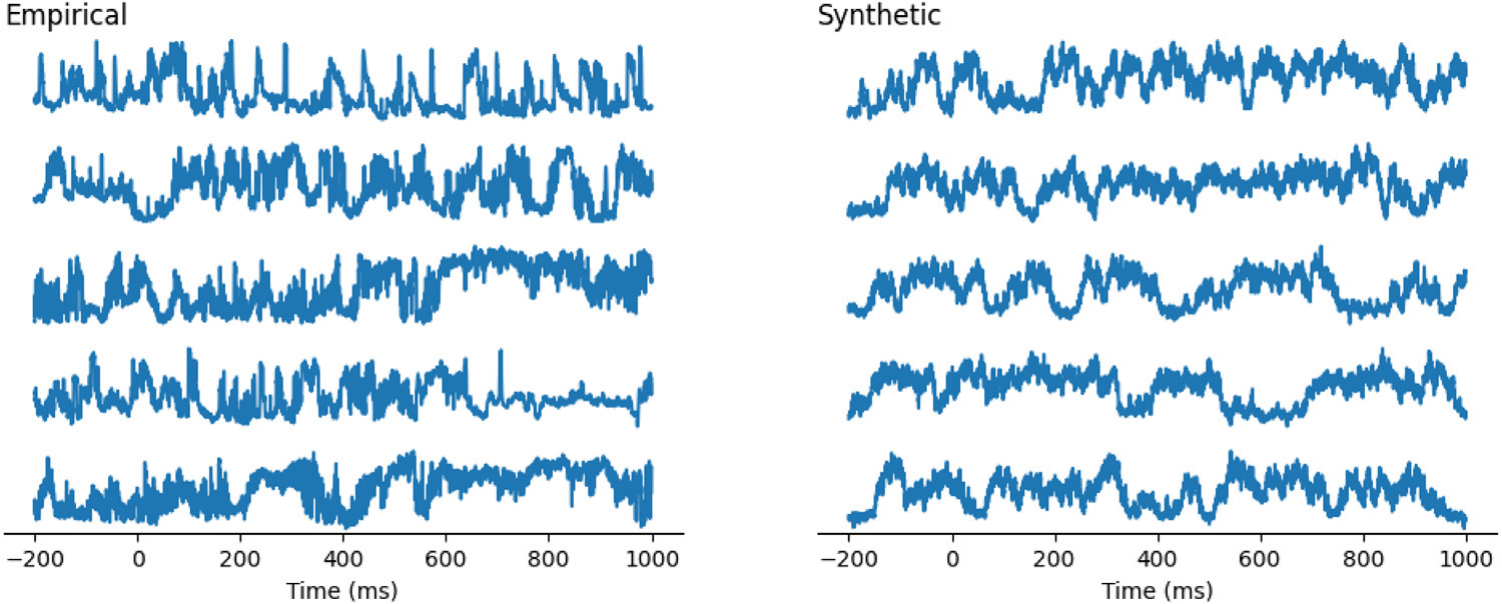
Empirical and synthetically generated EEG data.

We qualitatively evaluated empirical and synthetically generated EEG samples visually shown in Fig. 4. Further, we found that empirical and synthetic EEGs’ visuals indicated indistinguishable resemblances.

As for classification performances, a random forest classifier is employed to classify valence and arousal emotions. Generated synthetic data for male, female and mixed data samples are evaluated for empirical and augmented data separately. Overall, valence class performances overcome to arousal results. The augmented EEG had the highest mark with female data reaching up to 4.44% for valence, while it reaches 6.39% for arousal. Results show the correlation between sample size and accuracy is inverse, while sample size diminishes accuracy increases. This results supports the argument of inverse correlation proposed by Williams et al. (2023) [8]. Results for sample groups with arousal and valence classes are depicted in Table 8.

**Table 8:** Classification results for empirical and augmented data of three sample sizes.

Method	Valence (%)					Arousal (%)		
	Accuracy	Precision	Recall	F1-score	Accuracy	Precision	Recall	F1-score
Male Empirical	60.56	63.94	90.17	74.82	41.11	56.41	19.82	29.33
Male Augmented	60.28	63.66	90.6	74.78	40.83	55.7	19.82	29.24
Mixed Empirical	61.94	64.22	93.59	76.17	40.28	52.59	31.98	39.78
Mixed Augmented	63.33	65	94.44	77	42.22	56.25	28.38	37.72
Female Empirical	60.56	64.11	89.32	74.64	52.5	60	68.92	64.15
Female Augmented	65	65.79	96.15	78.12	58.89	63.03	80.63	70.75

Furthermore, an analysis of synthetically generated data is made to compare classification outcomes for three groups. Utilizing synthetic sample groups that are generated, the validation of synthetic data is assessed. In the context of classification, synthetic data is split into 80% training data and 20% test data for each group. The validation results and performance metrics are presented in Table 9. The outcomes show synthetic data as a separate dataset shows superior performances for male and female group while mixed group gives almost near results with empirical data.

**Table 9:** Classification results for synthetic data of three sample sizes.

Method	Valence (%)				Arousal (%)			
	Accuracy	Precision	Recall	F1-score	Accuracy	Precision	Recall	F1-score
Male Synthetic	65.5	68.19	60.85	64.31	60.17	62.21	56.12	59.01
Female Synthetic	70.33	73.93	64.76	69.04	71.08	75	65.09	69.69
Mixed Synthetic	58.64	59.6	54.61	57	59.42	60.22	56.45	58.27

**Fig. 5:**
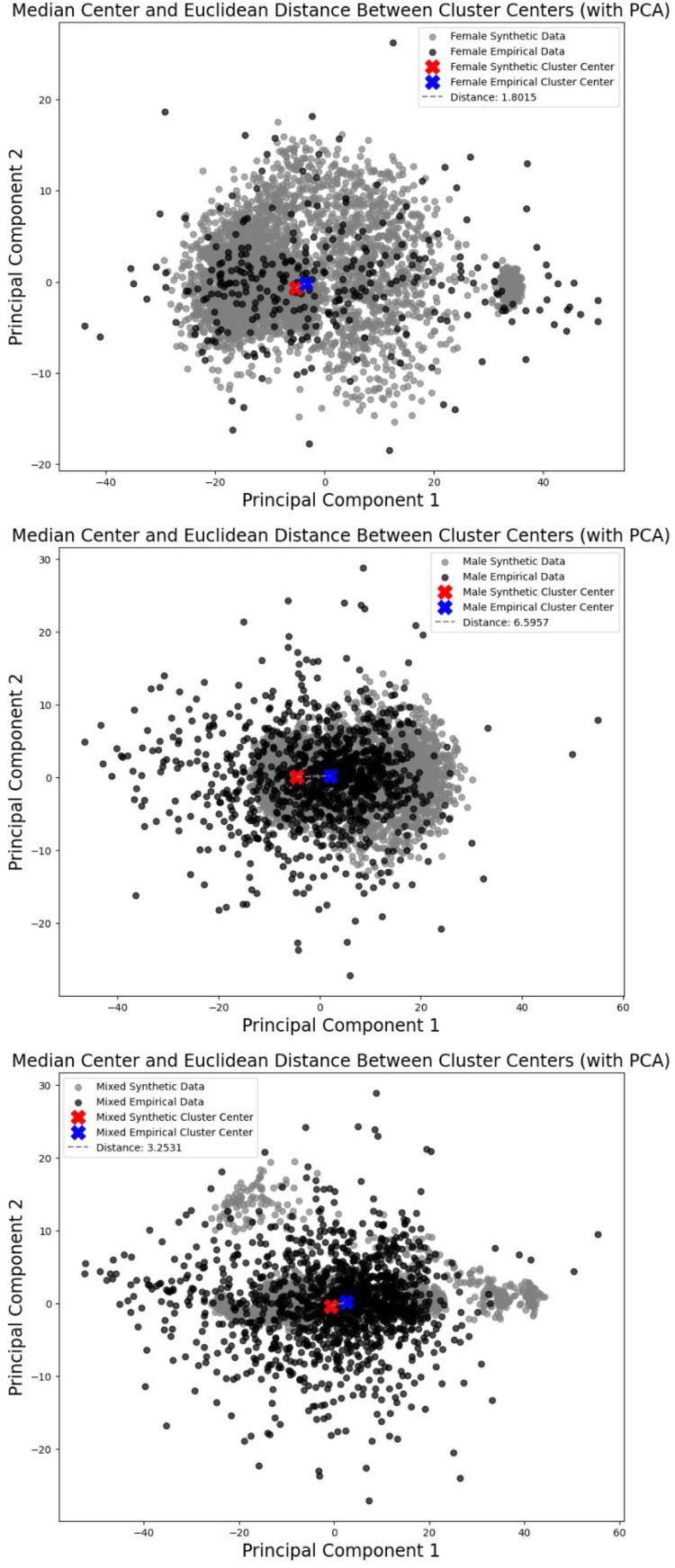
Euclidean distances between medians of three sample groups.

Additionally, a further validation is provided for EEGs generated synthetically by comparing the medians of empirical and synthetic EEG clusters via Euclidean distance. Initially, the dimensionality of empirical and synthetic EEG data is reduced to two components through the implementation of principal component analysis (PCA). The medians of each data cluster are subsequently computed. The formula for calculating the Euclidean distance between medians involves taking the square root of the sum of the squared differences between the two vectors. The data distribution and distances between the three groups are depicted in Fig. 5. Visually, synthetic EEGs exhibit similarities to empirical data concerning the outcomes of the three groups. The female group provides the shortest distance with 1.8, indicating a high degree of similarity between synthetic and empirical data, followed by the mixed group with 3.25, with the male group providing the greatest distance of 6.6.

In conclusion, overall findings showed that GAN-augmented EEG can enhance classification performance with random forest classifier. Data augmentation for EEG signals are evaluated using GAN method. The outcomes inform that synthetically generated EEGs can show resemblance and help to increase data size for more promising evaluations. The classification performances can be enhanced with augmented EEGs which open a way to improve performances of small datasets.

## Limitations

None.

## Ethics Statement

The informed consent was obtained from participants*.* The data acquisition was carried out in accordance with the Declaration of Helsinki, and the project was approved by Faculty of Computing, Engineering and Media at De Montfort University (CEM ID No G421051).

## CRediT authorship contribution statement

**Omer Faruk Kucukler:** Conceptualization, Methodology, Software, Data curation, Writing – original draft, Visualization, Investigation, Software. **Abbes Amira:** Conceptualization, Supervision, Writing – review & editing. **Hossein Malekmohamadi:** Conceptualization, Supervision, Writing – review & editing.

## Data Availability

EEG Dataset Collected During Energy Data Visualization Stimuli Presentation (EDAVIS) (Original data) (Mendeley Data).
